# A promising new tool for literacy instruction: The morphological matrix

**DOI:** 10.1371/journal.pone.0262260

**Published:** 2022-01-19

**Authors:** Melvin M. R. Ng, Peter N. Bowers, Jeffrey S. Bowers

**Affiliations:** 1 Chinese University of Hong Kong, Singapore, Singapore; 2 WordWorks Literacy Centre, Wolfe Island, ON, Canada; 3 University of Bristol, Bristol, United Kingdom; University of Windsor, CANADA

## Abstract

There is growing interest in the role that morphological knowledge plays in literacy acquisition, but there is no research directly comparing the efficacy of different forms of morphological instruction. Here we compare two methods of teaching English morphology in the context of a memory experiment when words were organized by affix during study (e.g., a list of words was presented that all share an affix, such as <doing>, <going>, <talking>, <walking>, etc.) or by base during study (e.g., a list of words was presented that all share a base, such as <doing>, <done>, <redo>, <undo>). We show that memory for morphologically complex words is better in both conditions compared to a control condition that does not highlight the morphological composition of words, and most importantly, show that studying words in a base-centric format improves memory further still. We argue that the morphological matrix that organizes words around a common base may provide an important new tool for literacy instruction.

## Introduction

Morphemes constitute the smallest units of meaning in an oral or written language, and in the case of English, include bases (e.g., <help>) that carry the main kernel of meaning in a word, and affixes (e.g. the prefix <un-> or the suffix <-ful>) that modify the meaning of the base (e.g., <unhelpful>). There is growing interest in the role that morphological knowledge plays in literacy acquisition in English. This interest is motivated by two general considerations. Firstly, English is a morphophonemic language with an orthographic system that evolved to represent both phonology and morphology. Indeed, as we describe below, the English spelling system prioritizes the consistent spellings of morphemes over phonemes. This provides a rationale for teaching morphology along with phonology and how they interrelate. Second, empirically, there is now ample evidence that morphological knowledge in young children predicts literacy outcomes [1], and growing evidence that morphological instruction improves literacy outcomes, especially for younger and less-abled readers [2, 3]. Despite these theoretical and empirical considerations there is almost no guidance for how to teach morphology in the classroom, nor research into how to best teach English morphology to children in order to improve reading, writing, and vocabulary [4, 5].

**Fig 1:**
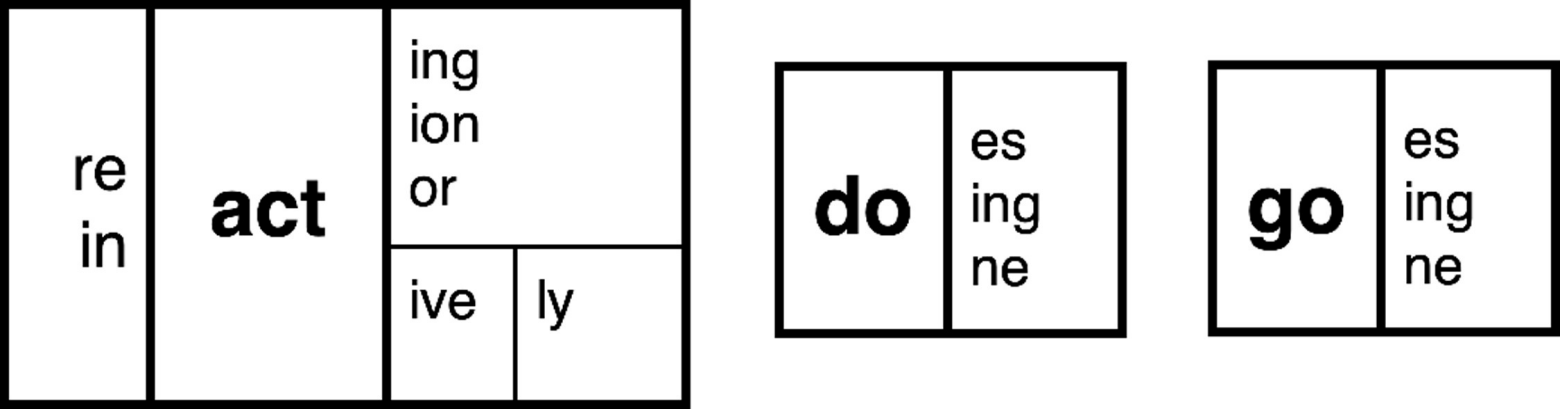
An example of a morphological matrix.

Here we compare two approaches of teaching morphology in the context of a memory experiment carried out with undergraduate students. The first was designed to mirror an affix-centric approach that focuses on teaching affixes one at a time. For example, students might be presented with a set of unrelated base words that all share a given affix in order to highlight the spelling, meaning, and grammatical role of the affix (e.g., presenting a list of words that all share the <-ing> affix such as <doing>, <going>, <talking>, <walking>, <playing>, etc.). This approach appears to be the most common guidance given to teachers [6]. The second was designed to mirror the base-centric approach used in Structured Word Inquiry or SWI [7, 8]. SWI teaches words in the context of morphological families composed of words that share a common base (e.g., <do>, <doing>, <redo> and <done>). A common tool of SWI is the morphological matrix, as depicted in Fig 1. We show that memory for morphologically complex words in a free recall task is better in both the affix- and base-centric morphological matrix conditions compared to a control condition that does not highlight the morphological composition of words, and importantly, demonstrate that studying words in a base-centric morphological matrix improves memory further still. We attribute these findings to the fact that memory, learning, and reasoning is best when information is encoded in an elaborative and organized manner [9, 10].

Our use of undergraduate students to assess the promise of different forms of instruction with children is not uncommon [11, 12]. The advantage of this approach is that it is more straightforward to assess the impact of different learning conditions in tightly controlled conditions. The obvious disadvantage, however, is that it is not certain that the results will extend to younger children in a classroom setting. Still, clear-cut findings obtained with adults can provide a strong motivation to explore similar interventions in children. And in this case, we take our findings to motivate further research into the morphological matrix as a teaching tool for reading instruction. Our findings may also help explain the recent disappointing results obtained in a large-scale study carried out with grade 5 students that adopted an affix-centric approach to morphological instruction [13].

The paper is organized as follows. First, we review the theoretical and empirical motivation for teaching morphology in order to improve various literacy outcomes including word naming, spelling, and vocabulary. Second, we briefly review different forms of morphological interventions, including affix-centric and various base-centric approaches that have been employed in intervention studies. Although there is evidence that morphological instruction is beneficial, there is no research to date directly comparing the efficacy of the different approaches. Third, we report two experiments that compare an affix-centric to a base-centric approach that uses morphological matrices. Finally, in the General Discussion, we consider the implications of our results for literacy instruction.

### The theoretical motivation for teaching morphology

It is widely claimed that the English spelling system follows an “alphabetic principle” according to which letters represent meaning via sounds, and where the grapheme-to-phoneme system is riddled with irregularities [14]. But in fact, English has a morphophonemic spelling system that evolved to jointly reflect meaning (through influences of morphology and etymology) and pronunciation (through representations of phonemes). This provides a key motivation for teaching children the important role that morphology plays in explaining not only the meaning-spelling connections between words, but also the spelling-pronunciation connections informed by morphology. Since the pronunciation of morphemes in English shifts across words, it is not possible to have a one-to-one relationship between phonemes and graphemes (e.g., the grapheme <o> maps onto different phonemes in the words <do> and <does>, but maintains consistent spelling of the base morpheme <do>). Writing systems like English with many ways of spelling the same phonemes are described as having a “deep” orthography. This contrasts with languages with “shallow” orthographies such as Italian and Spanish where grapheme-phoneme correspondences are largely one-to-one (highly consistent) [15].

To illustrate the morphophonemic organization of English spellings, consider the morphological families formed from the bases <act>, <do> and <go> in Fig 1. The important point to note is that the spelling of the base remains consistent across all members of the morphological families even when changes in pronunciation are observed (e.g., *actor* vs. *action*; *do* vs. *does*; *go* vs. *gone*). Or consider the <-ed> suffix. In a word like “painted” the pronunciation of the <-ed> suffix is syllabic, and thus needs the <e> grapheme to represent the vowel phoneme of that syllable. However, the <-ed> suffix is not syllabic in “jumped” or “played” and countless other words. In such words there is no pronunciation associated with that <e> grapheme. Technically, the <e> grapheme in the <-ed> suffix of these words represents a “zero allophone” /∅/ of the /ə/ phoneme that is pronounced in words like “painted”. This “zeroing” of a phoneme frequently occurs and it makes sense in the context of a morphemic family. For example, the <t> grapheme represents the /t/ phoneme in the “print” or “printing”, but it is zeroed when the <-s> suffix is added to form the word “prints” or “footprints”. The <t> grapheme in the base <rupt> (meaning “break”) can represent the /t/ phoneme in “bankrupt” or “disrupt”, the /ʃ/ in “disruption”, the /tʃ/ in “rupture” and it is zeroed when we add the <-cy> suffix to produce the word “bankruptcy.” It is important to emphasize that the above examples of morphological constraints on spelling are not cherry-picked; they are the norm.

**Fig 2:**
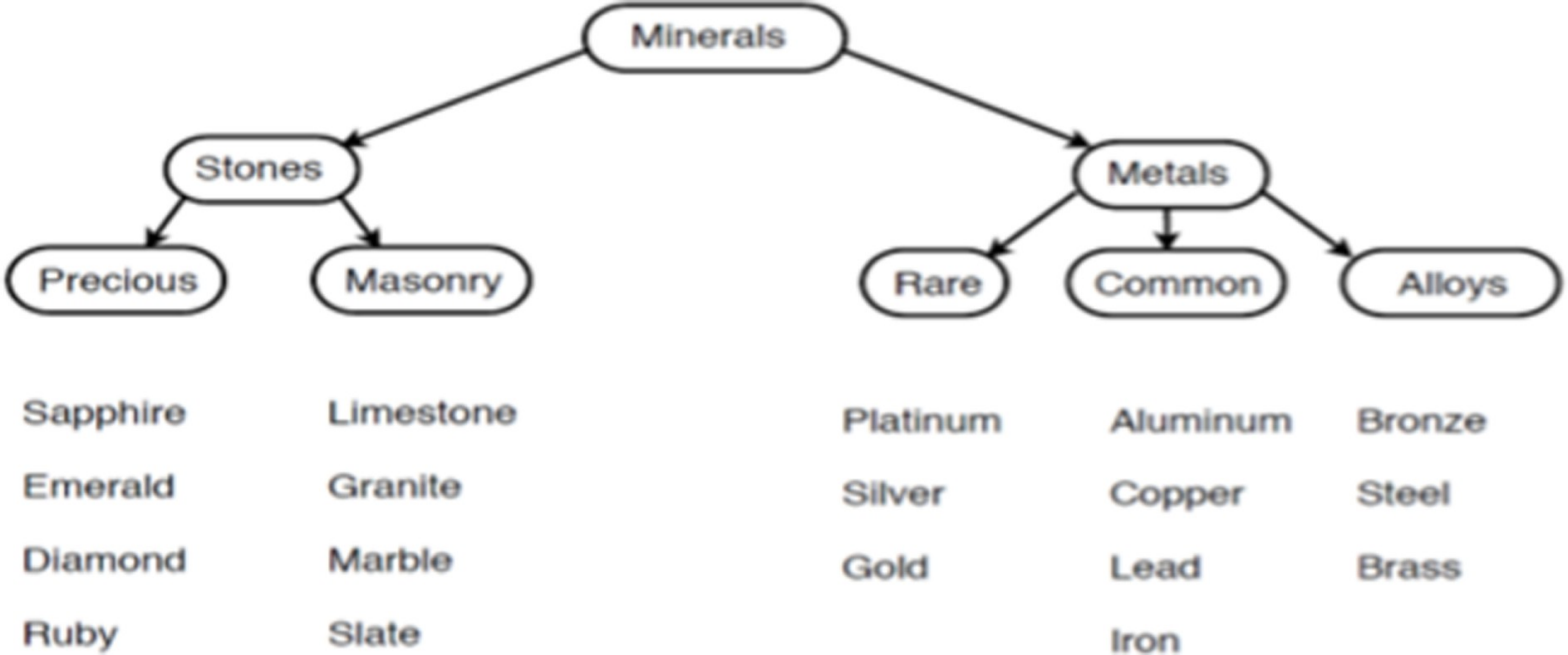
Hierarchy highlighting meaningful relations amongst words.

**Fig 3:**
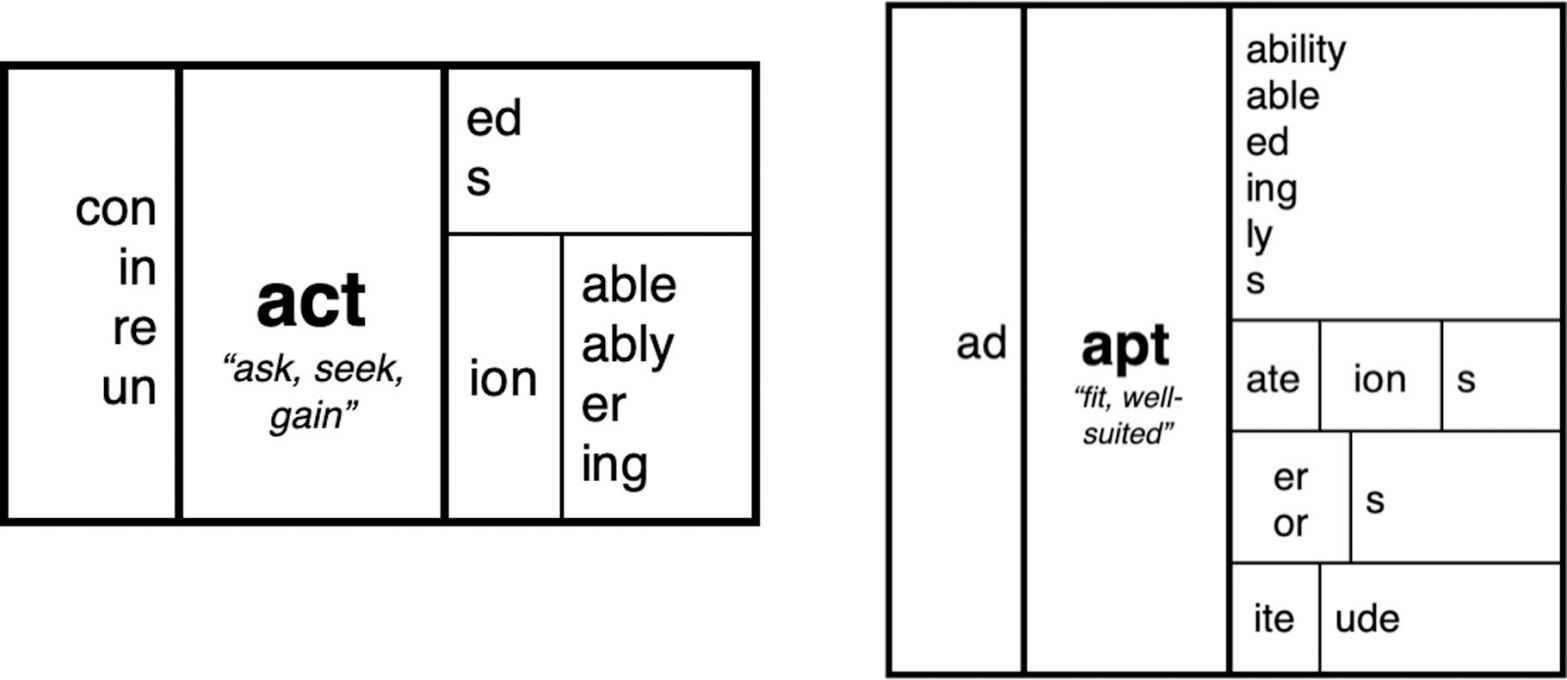
Morphological matrices highlighting membership in morphological families.

It should also be noted that there are two types of affixes, namely, derivational and inflectional. When derivational affixes are added to a base or a complex word, the result is considered a “new word,” or technically, a new lexical item (e.g., “help” vs “helpful”). By contrast, adding inflectional suffixes to a word results in what is considered different forms of the same word (e.g., “help” vs. “helps”). Technically, words like “help” and “helps” are considered the same lexical item. Inflectional suffixes mark grammatical relationships such as plural, past tense and possession, but unlike derivational suffixes, cannot alter the grammatical class of the words they construct [16]. This means that derivational affixes can dramatically change the meaning of the base (“cover” vs. “discover”) whereas the meaning shifts are minimal for inflectional affixes (e.g., “cover” vs. “covered”). Nevertheless, it is important to note that the phonological shifts that occur with derivations (“actor” vs. “action”) apply equally to inflections (“do” vs. “does”), as do spelling shifts. For example, the inflectional suffix <-ing> in “hopping” and “hoping” forces spelling changes in the base a result of suffixing conventions: i.e., doubling the <p> in the spelling <hopping> (hop(p) + ing—> hopping), and dropping the <e> in the spelling <hoping> (hope/ + ing—> hoping). An appreciation of the underlying logic of the English writing system has long motivated the hypothesis that reading instruction should emphasize both the phonological and meaningful regularities in word spellings [17–19]. Furthermore, as highlighted by Bowers and Bowers [8], this hypothesis is consistent with the finding that learning and memory is best when information can be encoded in a meaningful and organized manner [9, 10]. For example, Bower et al. [9] carried out a memory experiment in which words were organized within a hierarchy that highlighted the meaningful relations amongst the words, as depicted in Fig 2. Memory was much better in this condition compared to a condition that did not highlight these relations. In a similar way, morphological matrices (as depicted in Figs 1 and 3) highlight the meaning (and spelling) relations between words, and importantly, most words in English are members of morphological families. This raises the possibility that morphological matrices might be an effective way to learn the spelling, vocabulary, and pronunciation of most English words as well.

### The empirical motivation for morphological instruction

The linguistic analysis of the English writing system shows why morphology (and morphological matrices) might be relevant to literacy instruction, and memory research provides some theoretical motivation for emphasizing the meaningful organization of words. But is there any empirical evidence that morphological knowledge and morphological instruction benefits literacy?

Research has shown that prior to starting school, children already possess rich morphological knowledge as reflected in their language production and comprehension [20], and that untaught morphological knowledge is a good predictor of word naming, spelling, and vocabulary. For example, Kirby, Geier, and Deacon [20] found that morphological knowledge uniquely predicted reading speed, accuracy, decoding and comprehension in a sample of 182 Grade 3 students even after controlling for various other aspects like verbal and nonverbal intelligence, phonological awareness, naming speed and orthographic processing. Similarly, Deacon, Kirby and Casselman-Bell [21] found that morphological knowledge measured in Grade 2 was able to explain approximately 8% of the variance of Grade 4 spelling even after controlling for other aspects like verbal and nonverbal intelligence, phonological awareness, verbal short-term memory, and rapid automized naming. In the case of vocabulary, McBride-Chang and colleagues [22] provided evidence that morphological knowledge was able to predict vocabulary learning in English-speaking kindergarten and Grade 2 students. They found that morphological measures uniquely contributed to an additional 10% of the variance in vocabulary in either group upon controlling statistically for other measures like those of reading, phonological awareness and naming speed. For a recent review of the impact of morphological awareness on literacy see Duncan [1].

With regards to instruction, it has long been argued that morphology should play a more prominent role [17, 18, 22–24], but only in the past decade have a sufficient number of morphological intervention studies been carried out to support meta-analyses and systematic reviews. Goodwin and Ahn [2] reported a meta-analysis that showed moderate and significant improvements on a range of outcomes for children with learning disabilities, and Goodwin and Ahn [3] reported a meta-analysis that showed moderate and significant improvements on a range of outcomes for children from the general population. Similar conclusions were made in systematic reviews [4, 25, 26]. Most recently, in a meta-analysis of spelling interventions with children with dyslexia, morphological instruction was found to be effective, and just as effective as phonics overall in the early years of instruction [27]. Indeed, they found that morphological and orthographic instruction became more effective for children with spelling difficulties, whereas phonics instruction became less effective for struggling spellers, consistent with previous research on phonics, as summarized by Bowers [28].

### How is and how should morphology be taught?

Given these compelling reasons to include morphology as an ingredient to literacy instruction, it is striking how rarely morphology plays a role in reading, spelling, and vocabulary instruction in the classroom. This is highlighted by the fact that teachers know very little about morphology [29, 30]. To the extent that morphology is taught in schools there appears to be an emphasis on affix centric approaches, although the teaching guidelines we can find are all extremely vague. For example, in the US, “The Common Core State Standards for English Language Arts & Literacy in History/Social Studies, Science, and Technical Subjects” [31] mentioned morphology twice, writing that Grade 4 and 5 students should “use combined knowledge of all letter-sound correspondences, syllabication patterns, and morphology (e.g., roots and affixes) to read accurately unfamiliar multisyllabic words in context and out of context” (p 17), and with regards to vocabulary instruction writes “Use the most frequently occurring inflections and affixes (e.g., -ed, -s, re-, un-, pre-, -ful, -less) as a clue to the meaning of an unknown word.” (p. 27). Similarly, in the UK National Curriculum for children in Key Stage 1–2 (ages 5–11), the main information regarding morphological instruction is found in tables in the document that provide some general guidance for affix centric instruction (e.g., page 13, 46–52). There is also brief mention of a more base-centric approach, with the following included in another table describing instruction for Year 3 students: “Word families based on common words, showing how words are related in form and leaning [for example, solve, solution, solver, dissolve, insoluble]” (p. 66). An affix-centric approach is likewise encouraged in countries such as Hong Kong, where teachers are advised to focus on affixes from Secondary levels 1–3 [32], and in Singapore, where it is recommended that the concept of affixes be introduced from Primary 1 onwards [33]. In all cases, English teachers are left to their own devices to teach morphology as they see fit, with only general guidance in affix-centric (and on occasion base-centric) instruction.

While less common than affix-centric approaches, there are also different forms of base-centric instruction reported in the literature. For example, “Words Their Way” [34] is a popular program which targets more advanced derivational morphology in upper elementary than many resources. This approach begins with activities for sorting words around affixes, then moves to sorting words around common bases. For example, students are asked to sort words around what they describe as a Latin Root <dic> for “speak, say” (such as <verdict>, <dictate>) or <vis> for “see” (such as <vision> or <revise>). Because the to-be-sorted words are presented to students this is called morphological ‘recognition tasks.’ There are also production base-centric tasks where children create new words that are part of a morphological family [35]. Structured word inquiry [7, 8] involves both recognition and production: when students are asked to analyze the words in a matrix they are engaging morphological recognition and when they create their own matrices from a base, they are engaging in production. Promising results have been obtained with all of these approaches, including with young children in Grade 1 [36, 37], but there is no evidence whether some base-centric approaches are more effective than others.

Even the more basic contrast between affix- vs. base-centric morphological instruction has received little attention. The research literature does include studies that have specifically focused on affixes and others that have focused on affixes and bases. For example, P. Bowers et al. [4] reviewed 22 intervention studies and found that whereas all targeted affixes only 8 targeted bases or stems. The authors did not attempt to compare the effectiveness of these two different approaches because of the variability of the studies, and neither did Goodwin and Ahn [3]. Interestingly, Reed [26] concluded that stronger effects were associated with instruction focused on root (base) words compared to affixes alone in a review of morphological interventions, but this was based on a small (and variable) set of studies making any conclusion insecure.

### The present study

Here we report two experiments that directly compare an affix-centric approach to a base-centric approach using morphological matrices (as in Figs 1 and 3). In both experiments, participants studied a set of morphologically related words for a subsequent memory task when groups of words were organized by a common affix (using an affix-centric morphological matrix) or base (the base-centric morphological matrix used in SWI), or when randomly intermixed with no reference to morphology (control condition). Given that learning and memory is best when information can be encoded in a meaningful and organized manner [9], we predicted that memory performance would be better in the two morphological conditions compared to the control condition. Our critical prediction, however, is that organizing words by base in a base-centric morphological matrix will support better memory performance than organizing words by affix, and that this would extend to both familiar (e.g., mistake) as well as unfamiliar (e.g., misbalance) morphologically complex words.

## Experiment 1

### Method and results

#### Participants

A total of sixty-two English-speaking participants (mean age = 21.3 years, SD = 2.43 years; 30.6% were males) studying various majors at a large British university participated for either course credit or payment. Participants reported normal language abilities and normal or corrected-to-normal vision. No participants were excluded as all of them were able to successfully complete the task. Participation occurred with informed consent and the experimental protocols were approved by the Institutional Review Board.

This sample size can be justified on the assumption of a large effect size of d = 1.0 for the base-centric condition over the affix and control condition. Consistent with this, Bower et al. [9] reported memory performance over three times higher in the organized vs. disorganized memory condition with zero overlap between performance in the two groups (the effect sizes were so large that statistics were not even reported). In addition, in their review of generative learning strategies, Fiorella and Mayer [10] found that matrices (not morphological matrices) that organized to-be-learned information in a meaningful manner were the most effective method for improving learning in a classroom setting, with a median effect of d = 1.07 across eight studies (findings discussed in more detail in the General Discussion). Although there are important differences with our work, these findings lead us to predict large effect sizes. Based on G-Power analysis [38], an effect size of d = 1.0 with an alpha of 0.05 and power of 0.8, requires 17 participants per group. Accordingly, our sample size should be adequate to detect an effect size around this magnitude.

#### Design and materials

Participants were randomly assigned to one of three study conditions: A control condition (n = 21) in which words were presented in lists without any form of organization, an affix-centric condition (n = 20) in which words with different bases were grouped by a common affix in a matrix format, or a base-centric condition (n = 21) in which a morphological family of words that all shared a common free base were presented in a morphological matrix much like that used in SWI. We selected 40 morphologically complex words constructed from 10 base words and 10 affixes, half of which comprised either a base word combined with a prefix (e.g., un + cover → uncover), and the other half, a base word combined with a suffix (e.g., count + er → counter). These words were chosen from online dictionaries such as Lexico [39], Merriam-Webster [40], Collins [41] and online resources such as “Word Searcher” [42]. The words were selected by first determining some of the most commonly used prefixes and suffixes. This was followed by a selection process where base words were chosen based on whether they could form plausible morphologically complex word forms using the prefixes and suffixes that were selected in the previous step. In the base-centric study condition each base word was combined with two prefixes and two suffixes, whereas in the affix-centric condition these same affixes were combined with four bases (see S1 Appendix which contains all the appendices).

In the control condition, words were organized in lists of 4 randomly selected words from the pool of 40 words. Accordingly, all participants studied 10 lists each composed of four morphologically complex words, and all participants studied the same set of 40 words. Note, not all affixes were studied with all bases (e.g., the prefix <dis> was studied with the base <charge> but not <cover>), and accordingly, participants needed to remember which affixes were combined with which bases during the study phase. It is also the case that the spelling of some bases changed when adding an affix (e.g., charge/ + ing → charging), and that some of the morphologically complex words were highly familiar (e.g., mistake) and others not (e.g., misbalance). Regardless of whether such words are familiar or not, a general sense of what these words must mean can be deduced when the reader is familiar with the morphemes constructing the words. The word frequency of the 40 morphologically complex words according to the English Lexicon Project [43] are listed in S1 Appendix, which contains all the appendices. Each participant studied and recalled the 40 words twice.

#### Procedure

**Fig 4:**
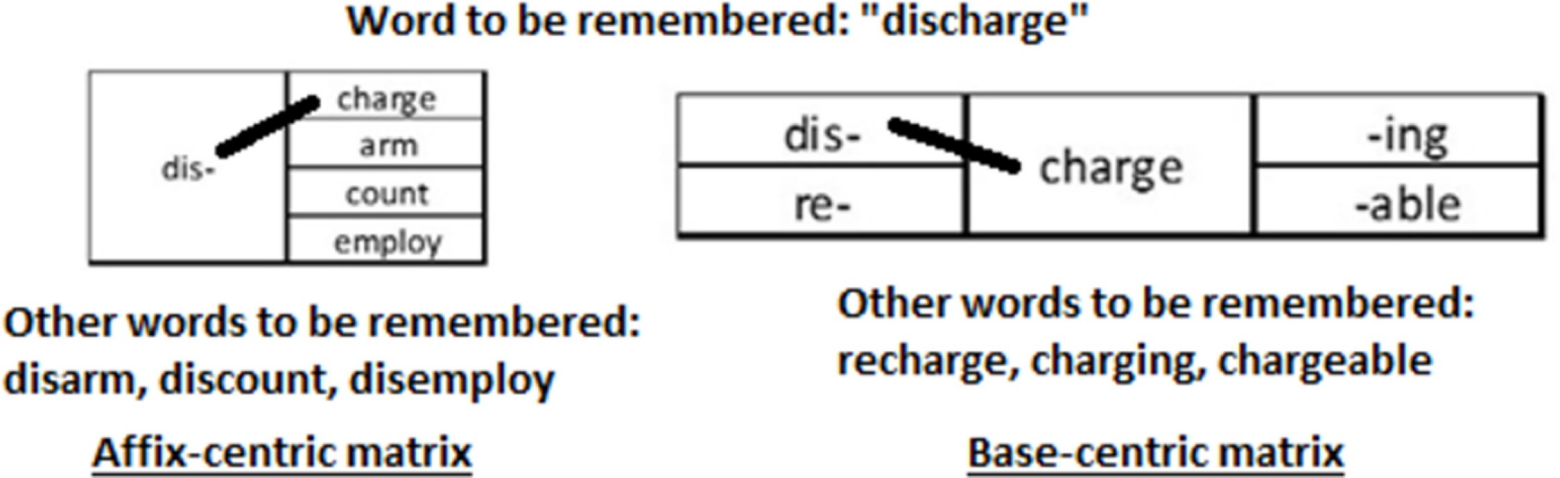
How the matrices were used to form the to-be-remembered word.

Participants sat in front of a computer and were tested in groups of 1–6 with stimuli presented using PsychoPy 3 [44]. Participants were instructed to remember the words presented on the screen. A practice session was conducted to familiarize participants with the general flow of the experiment. During this practice session, participants were given examples of how the stimuli would be presented in the experiment, as well as explicit instructions and a demonstration as to how to combine the affixes and bases in the affix- and base-centric conditions to form the to-be-remembered words. An example of this is shown in Fig 4. For the base-centric matrices, participants were instructed to study the base combined with all the prefixes and the base combined with all the suffixes (e.g., discharge or charging), but not the base combined with a prefix and a suffix (e.g., discharging).

As the participants in the study were university-level students and experienced readers, the basic principle of dropping the final “e” in some of the words was not elaborated on, and in fact, such an error was absent in the responses made later on. Each participant was given 3 seconds to remember each word, with each stimulus list or matrix (composed of four words) presented for a total of 12 seconds. The order in which the lists and matrices were presented was randomized. After all lists or matrices were presented participants wrote down as many words that they could remember on a blank slip of paper. Participants were free to write down the words in any format that they chose, and while not explicitly told to do so, some chose to represent their answers in the matrices that had previously been presented. This slip of paper was then collected from the participants. The experiment was then repeated using the same materials and procedure without the practice session. The order in which the lists and matrices were presented were again randomized. This provided two sets of recall data in each condition, and it provided an assessment of whether the different methods of organizing words support greater or smaller differences with increased practice [9].

### Results and discussion

**Fig 5:**
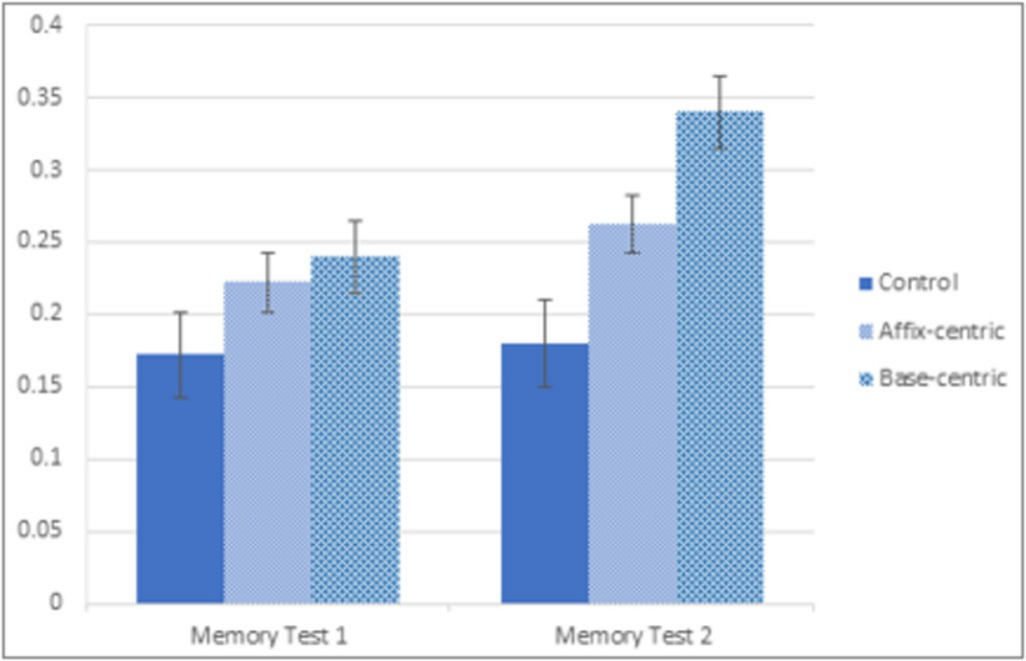
Mean recall scores for each group in each memory test for Experiment 1 (error bars indicate 1 standard error above and below the mean).

The mean recall scores in the three study conditions and two recall tests are summarized in Fig 5, with the y-axis being the proportion of words correctly recalled. A 3 x 2 between-within mixed ANOVA was conducted on the number of words correctly recalled, with Study Condition as the between factor (Control, Affix-centric and Base-centric) and Test Iteration as the within factor (Memory Test 1, Memory Test 2). Only words from the study phase that were correctly spelt were considered correct (erroneously recalled words and those that were misspelt were coded as incorrect). A main effect of test iteration was observed, with participants more likely to recall more words in the second compared to the first test, F1(1, 59) = 124.32, MSE = .336, p < .001; F2 (1,39) = 55.04, MSE = .315 p < .001. More importantly, we observed a main effect of study condition, F1(2, 59) = 16.37, MSE = .197, p < .001; F2 (2, 78) = 38.07, MSE = .463, p < .001, with both the affix-centric (24.2% recall) and base-centric (29.0% recall) conditions supporting better recall than the control condition (17.6% recall). We also observed a significant interaction between study condition and test iteration, F1(2, 59) = 6.29, MSE = .017, p < .005; for the item analyses, Mauchly’s test indicated a violation in the assumption of sphericity, χ^2^(2) = 14.136, p = 0.001, and so the Hyunh-Feldt correction was applied, F2(1.58, 59.51) = 8.199, MSE = .074, p < .005. Taken together, this reflected the greater memory performance in the base-centric condition in the second memory test. The planned comparison between the affix- and base-centric study conditions was significant, t1(39) = 2.17, p = .036; t2(39) = 2.57, p = .014, corresponding to a medium sized effect (Cohen’s d = 0.68), with a null effect observed in memory test 1, t1(39) = .679, p > .05; t2(39) = .761, p > .05 (Cohen’s *d* = 0.22), and a robust effect in memory test 2, t1(39) = 2.96, p = .005; t2(39) = 3.128, p < .005 (Cohen’s *d* = 0.95). Together these findings highlight the value of organizing words into their morphological components, and the added value of presenting words in a base-centric morphological matrix.

The pattern of errors that is summarized in Table 1 also highlights the impact of the morphological study conditions. We categorized errors into four sub-categories: Recombined Errors that reflected the correct recall of an affix and a base but recombining them into a non-studied word, partial errors that reflected the correct recall of an affix or a base but not both (i.e., a correctly recalled base or affix is paired with an affix or base outside of the study list), unrelated errors that reflected recalled words that included neither a studied based or affix, and spelling errors. For examples of some common errors, please refer to S1 Appendix. All error proportions were calculated with the total number of words presented as the denominator. As is clear from Table 1, the rate of recombined errors was much greater in the two morphological study conditions, leading to somewhat higher error rates in the two morphological conditions. Note, these recombined errors reveal that the participants were using morphology to inform their memory responses. In the context of a memory experiment, the increase in recombined errors might be considered a negative. But in the context of assessing the overall impact of the morphological encoding conditions on memory and learning more generally (where all morphological forms should be learned, not just studied words), these errors might be considered further evidence that the two morphological study conditions are more effective (reflecting the learning of new morphologically complex words). In Experiment 2 we redesigned the study lists in an attempt to reduce the recombined errors in order to get a more straightforward assessment of the benefits of encoding words in the morphological conditions.

**Table 1:** Error type (%) across study conditions for Experiment 1.

**Test 1**					
Condition	Overall	Recombined	Partial	Unrelated	Spelling
Control	10.71	3.87	0.50	6.35	0.00
Affix-Centric	13.66	12.04	0.12	1.50	0.00
Base-Centric	13.19	11.92	0.23	1.04	0.00
**Test 2**					
Condition	Overall	Recombined	Partial	Unrelated	Spelling
Control	11.41	5.46	0.30	5.56	0.10
Affix-Centric	14.47	13.89	0.23	0.35	0.00
Base-Centric	16.44	14.35	0.35	1.74	0.00

## Experiment 2

In order to reduce the incidence of errors we developed a new set of words in which eight bases and eight affixes were factorially combined (all affix-base combinations were studied), making a total of 64 words to recall. In this way, if a participant remembers a base and an affix correctly, he or she will not incorrectly combine them to produce a recombined error. Again, we anticipate that participants will use their morphological knowledge to improve memory performance in the two morphology conditions, and the critical question is whether the base-centric study condition improves memory performance more than an affix-centric condition.

### Method and results

#### Participants

A total of sixty-four English-speaking participants (mean age = 24; 28% were males) studying various majors at a large British university participated for either course credit or payment. This new set of participants was drawn from the same pool of participants as Experiment 1. Participants reported normal language abilities and normal or corrected-to-normal vision. No participants were excluded as all of them were able to successfully complete the task. Participation occurred with informed consent and the experimental protocols were approved by the Institutional Review Board. Once again, participants were assigned to either the control condition (n = 20), the affix-centric condition (n = 22) or the base-centric condition (n = 22).

#### Design and materials

A total of 8 free base words and 8 affixes were chosen, and all the base words chosen were able to form legal permutations with all affixes. These words were chosen from the same online resources that were used in Experiment 1. This allowed us to factorially combine all bases and affixes during study so that participants could no longer make Recombined Errors as was observed in Experiment 1. In total, 64 morphologically complex words were formed, half of which comprised either a base word combined with a prefix (e.g., re + value → revalue), and the other half, a base word combined with a suffix (e.g., value + s → values). Once again, the affix and base sometimes composed a more familiar word (e.g., revalue) and in other cases an unfamiliar word (e.g., <disvalue>). These words were grouped either by affixes (for the affix-centric condition) or their bases (for the base-centric condition) to form lists or matrices of 8 words each. Lists of 8 randomly selected words from the pool of 64 words were also created for the control condition. In total, 8 lists or matrices were created for each condition, with each participant studying all 64 words. For full details of the lists, matrices and words used, please refer to S1 Appendix.

#### Procedure

As above, participants were seated in front of a computer, tested in groups of 1 to 6, and asked to remember words that were to be presented to them on the screen. A practice session similar to that which was conducted in Experiment 1 was conducted to familiarize participants with the general flow of the experiment. Each participant was given 3 seconds to remember each word, with each stimulus list or matrix (composed of eight words) presented for a total of 24 seconds. The order in which the lists and matrices were presented was randomized. After all lists or matrices had been presented, participants were asked to write down as many words that they could remember on a piece of paper, which was collected from the participants once they were done with this portion of the experiment. The experiment was then repeated using the same materials and procedures, without the practice session.

### Results and discussion

**Fig 6:**
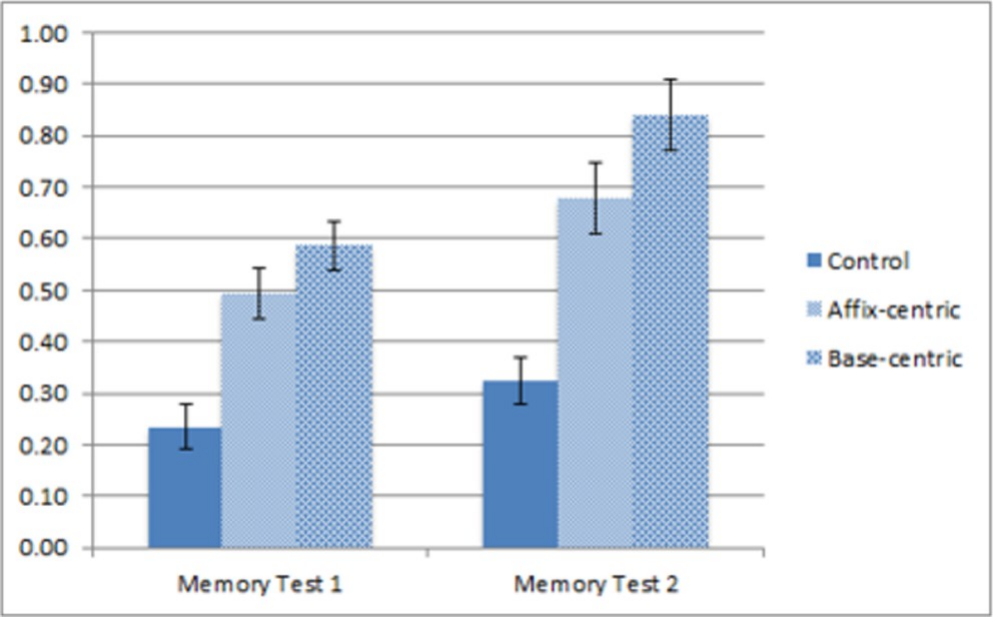
Mean recall scores for each group in each memory test for Experiment 2 (error bars indicate 1 standard error above and below the mean).

As before, only words from the study phase that were correctly spelt were considered correct, and the mean recall scores across conditions are summarized in Fig 6. A 3 x 2 between-within mixed ANOVA showed a main effect of test iteration, with participants performing better in the second compared to the first test, F1(1, 61) = 92.94, MSE = .984, p < .001; F2(1,63) = 331.22, MSE = 2.981, p < .001, and a main effect of study condition, F1(2, 61) = 41.609, MSE = 2.064, p < .001; F2 (2, 126) = 506.88, MSE = 6.659, p < .001, with both the affix-centric (58.7% recall), and base-centric (71.3% recall) condition supporting better recall compared to the control condition (28.0% recall). And again, a significant interaction was also found between test iteration and study condition, F1(2, 61) = 6.72, MSE = .071, p < .005; F2 (2, 126) = 24.75, MSE = .191, p < .001, reflecting the greater improvement in the base-centric compared to affix-centric conditions in the second memory test. The planned comparison between the affix- and base-centric conditions was again significant, t1(42) = 2.93, p = .005; t2(63) = 11.01, p < .001, corresponding to a large effect size (Cohen’s d = 0.88), with the contrast between the base- and affix-centric conditions failing to achieve significance in memory test 1 for the participant analyses, t1(42) = 1.67, p = .102 and significant in the item analyses, t2(63) = 5.78, p < .001 (Cohen’s *d* = 0.52), and robust in memory test 2 for both participant and item analyses, t1(42) = 2.559, p = 0.014; t2(63) = 11.76, p < .001 (Cohen’s *d* = 0.79).

Furthermore, as expected, the elimination of the Recombined Errors (due to fully crossing bases and affixes) resulted in much reduced overall error rates in the two morphological conditions. Indeed, the overall recall performance was much higher in Experiment 2 (52.5%) compared to Experiment 1 (28.3%), with recall rates most improved in the base-centric morphological matrix condition. The pattern of errors for experiment 2 is summarized in Table 2.

**Table 2:** Error type (%) across study conditions for Experiment 2.

**Test 1**					
Condition	Overall	Recombined	Partial	Unrelated	Spelling
Control	7.99	0.00	0.23	7.70	0.06
Affix-centric	4.33	0.00	0.36	3.05	0.92
Base-centric	2.58	0.00	1.14	1.20	0.24
**Test 2**					
Condition	Overall	Recombined	Partial	Unrelated	Spelling
Control	8.28	0.00	1.10	6.77	0.41
Affix-centric	3.69	0.00	0.50	2.06	1.14
Base-centric	1.50	0.00	0.48	0.30	0.72

**Fig 7:**
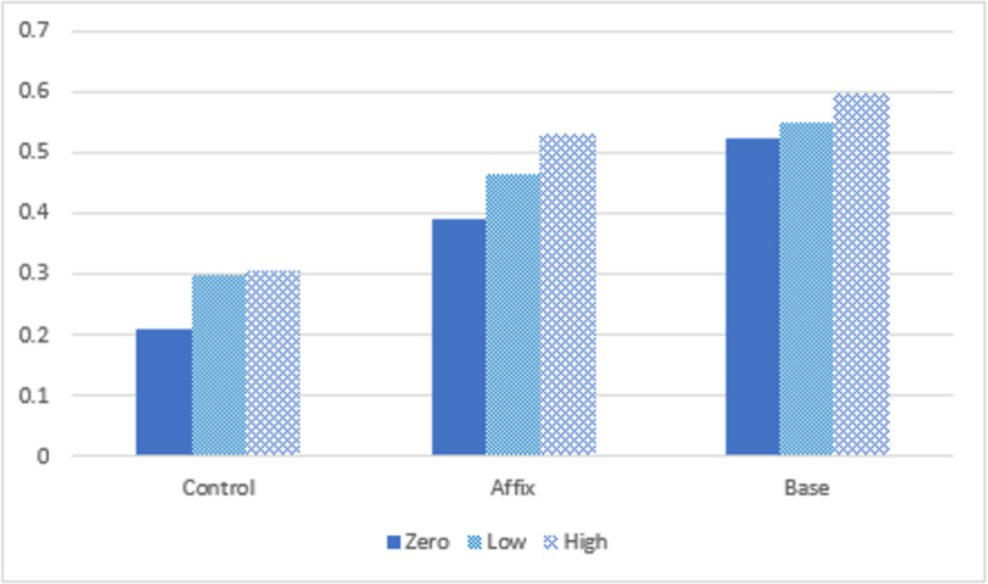
The overall memory results in terms of proportion of words recalled in the control, affix, and base conditions across both Experiments 1 and 2 for zero frequency, low frequency and high frequency words.

It is worth noting that we obtained the same pattern of results for the familiar and unfamiliar morphologically complex words in both in Experiments 1 and 2. The frequencies of the 40 morphologically complex words from Experiment 1 and 64 morphologically complex words from Experiment 2 were assessed using the English Lexicon Project [42] and were organized into a high-frequency category (43 words that all had a frequency of over 10 per million), low frequency category (38 words that all had a frequency between 10 and 0 per million), and zero frequency condition (32 words that did not occur amongst ~131 million words analyzed in the English Lexicon Project). Fig 7 summarizes the overall memory results in the control, affix, and base conditions across the two experiments for the three frequency conditions. The similar pattern of results obtained across the three frequency conditions shows that the memory advantage in the base-centric condition extends to low-frequency and novel words. This is consistent with the hypothesis that the base-centric morphological matrix is a useful tool for learning new words.

**Fig 8:**
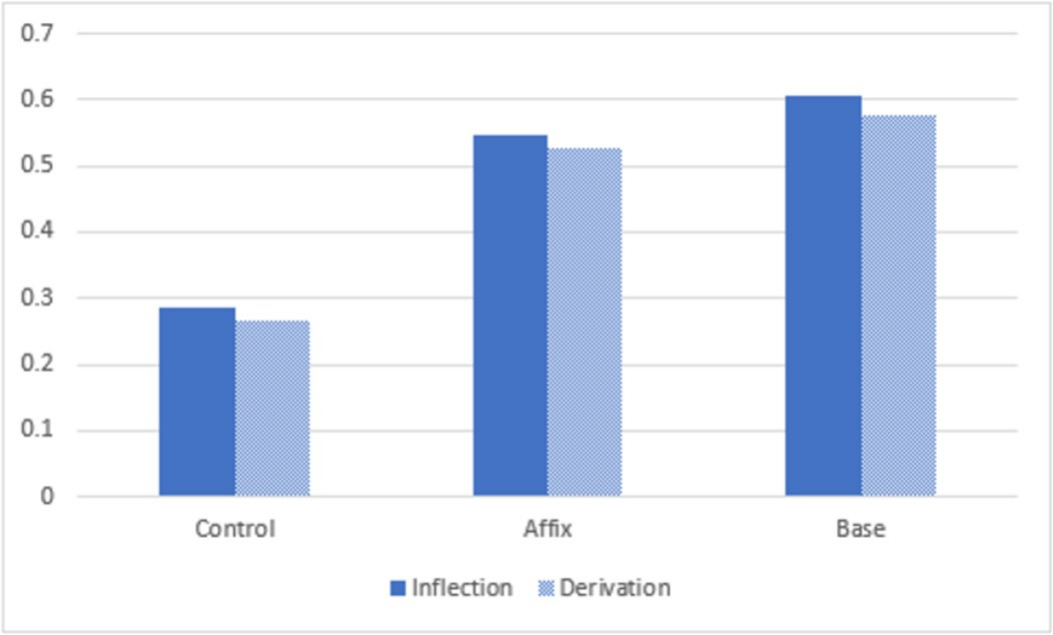
The overall memory results in terms of proportion of words recalled in the control, affix and base conditions across both Experiments 1 and 2 for inflections and derivations.

Another point worth noting is that the same pattern of results was obtained for inflections and derivations in both Experiments 1 and 2 (see Fig 8). This demonstrates the memory advantage in the base-centric condition extends to both inflectional and derivational words.

## General discussion

In two experiments we found that memory for a set of morphologically related words was better when the study conditions highlighted the morphological composition of words compared to a control condition that ignored this structure. The critical finding, however, is that memory was best when words were studied in a base-centric manner using morphological matrices that organized words into morphological families compared to a closely matched condition in which the same set of words were organized by affixes in matrices. As noted above, morphological instruction plays a minor role (at best) in literacy instruction in schools at present, and when morphology is considered, affix-centric approaches tend to be adopted. Our findings are important because they motivate the introduction of a new tool for morphological instruction with relevance to teaching spelling, vocabulary, and word naming. More generally, our finding lends some support to a wide range of base-centric methods that have been developed over decades but have not been widely adopted in schools [35].

Why did the morphological matrices improve memory outcomes? We take our findings to be closely related to the earlier finding from Bower et al. [9] who observed improved memory when a set of semantically related words were organized in a semantic hierarchy. This organization helped participants generate a retrieval plan that in turn improved memory performance. For example, in the illustration depicted in Fig 2, participants already knew many examples of gemstones and metals and could thus retrieve instances of gemstones and metals from background knowledge and then decide if the generated item was on the study list. The morphological matrices may have provided a similar benefit to memory retrieval strategies in our experiments given our participants likely already knew the meanings of the base words and affixes they had studied, and accordingly, they may have used their background morphological knowledge to generate plausible responses and then decide if the word was included in the study list.

Providing a context in which a learner can generate a retrieval plan may not only be useful for retrieval itself, but also the retrieval plan may improve the encoding of the to-be-remembered materials through “generative learning” that involves organizing and integrating new information with prior knowledge in order to improve the learning [10]. A classic finding in memory research is the “generation effect” in which generating a solution to a problem during the study phase of a memory experiment leads to better retention than being provided the answer to the problem at study [43, 45, 46]. For example, participants were better at remembering the word pair “rapid-fast” if they generated the word “fast” from the cue “rapid-f” during the study phase compared to simply studying the word pair “rapid-fast” [47]. Importantly, organized retrieval and generation effects may interact. For example, the memory advantage of organized vs. disorganized study condition in the above Bower et al. [9] study became larger following repeated memory tests, suggesting that an organized retrieval plan at memory test 1 improved the encoding of the words that in turn improved performance in memory test 2. We found a similar pattern here, with the advantage of the affix-centric and base-centric matrix conditions becoming stronger with repetition.

One common tool for generative learning that is especially relevant is a *graphic organizer* in which key concepts are arranged in meaningful format such as a matrix. Indeed, of all the generative learning strategies reviewed by Fiorella and Mayer [10], a matrix organizer was found to be the most effective with a median effect of d = 1.07 across eight studies. In the case of the morphological matrix, the content being arranged meaningfully just happens to be the orthographic structures of morphologically related words. Generative learning may help explain the improved performance of the base-centric compared to the affix-centric condition given that the base rather than the affix constitutes the core meaning of a word so that deeper semantic links can be developed when organizing words by base. It is also consistent with our finding that the memory advantage in the base-centric matrix condition extended to novel morphologically complex words, a case in which novel words can be organized and integrated with the familiar morphemes to make sense of them.

Of course, there is a large gulf between teaching morphology to children in the classroom and our memory experiment carried out on undergraduate students. Clearly further research is needed to determine whether the memory advantage we observed in the morphological base-centric matrix condition will translate into more effective morphological instruction in the classroom and under what conditions. Similarly, it is unclear from the current memory results whether the matrices would be best suited for teaching word naming, spelling, vocabulary, or some combination of these skills. But none of this undermines the main goal of the current research, namely, to provide a tightly controlled study that directly compares the efficacy of encoding words in an affix- and base-centric manner. We think our findings provide a strong motivation to explore the conditions under which morphological matrices are effective for instruction in children and highlight the value of base-centric instruction.

Indeed there are good reasons to hypothesize that our results are relevant to learning in children. Perhaps the most basic point is that the benefits observed in the morphological base-centric matrix condition reflect a fundamental property of memory and learning that applies to both adults and children; namely, it helps to study and recall information in a meaningful and organized manner. More directly relevant, there is some preliminary evidence that the morphological base-centric matrix does help literacy instruction in children. For example, Bowers and Kirby [7] reported that a Structured Word Inquiry study that included the morphological matrix improved vocabulary learning in grade 4 and 5 students. Similarly, Devonshire and Fluck [43] showed benefits in spelling for Year 4 and 5 students in England (ranging from age 7–9) following an intervention that included morphological matrices, and Devonshire et al. [36] reported benefits in spelling and naming aloud words in a Year 1 and 2 intervention (children aged between 5–7) using matrices. These studies all used a range of tools in addition to the morphological matrices, and the current study suggests that the matrix per se played a role in these results. It is interesting to contrast the study by Bowers and Kirby [7] with a recent large intervention by Foorman et al. [13] that adopted an affix-centric approach to teaching morphological knowledge. Both studies were concerned with improving vocabulary knowledge, and both studies targeted similar aged children, although the Foorman et. al study [13] included 200 minutes more instructional time and addressed far less content. Whereas Foorman et al. failed to observe any improvements in children inferring the meaning of novel words, Bowers & Kirby found children were better at defining words they had not been exposed as long as that word shared a base with a word they had been exposed to. The current study suggests that the use of the base-centric morphological matrix in the Bowers & Kirby study [7] may have contributed to the better outcomes.

In sum, our findings highlight that encoding words in a base-centric condition leads to better memory than a closely matched affix-centric condition. These findings suggest that that the morphological base-centric matrix may be a useful tool for morphological instruction, and support base-centric approaches to morphological instruction more generally. We hope our findings motivate future research that characterizes the conditions that morphological matrices are most useful for reading instruction and motivate more research into the Structured Word Inquiry method that uses the morphological base-centric matrix along with various other tools to not only teach about morphology, but also grapheme-phoneme correspondences in a meaningful context.

## Supporting information

S1 Appendix Contains all the appendices. (DOCX)
