# Supplementary material for: A promising new tool for literacy instruction: The morphological matrix
Source: PLoS One. 2022 Jan 19;17(1):e0262260. doi: 10.1371/journal.pone.0262260 (PMC8769298; doi:10.1371/journal.pone.0262260)
Supplement: S1 Appendix — (DOCX) [file pone.0262260.s001.docx]

**Appendix 1 Stimuli for Experiment 1**

Affix-centric condition


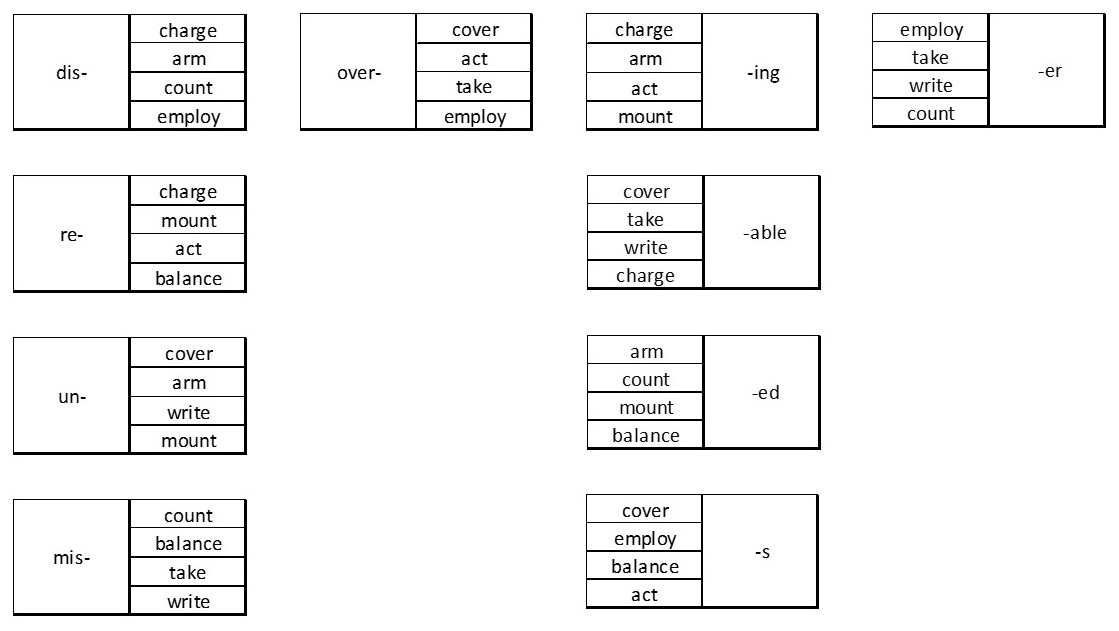


Base-centric condition


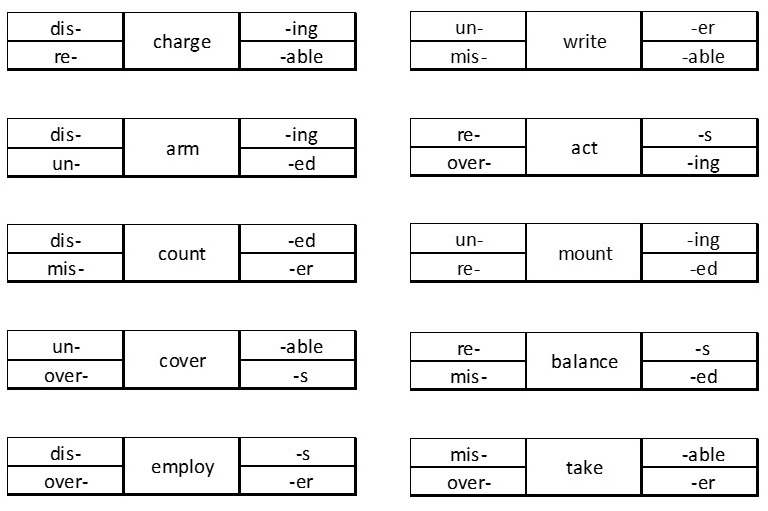


**Appendix 2 Stimuli for Experiment 2**

Affix-centric condition


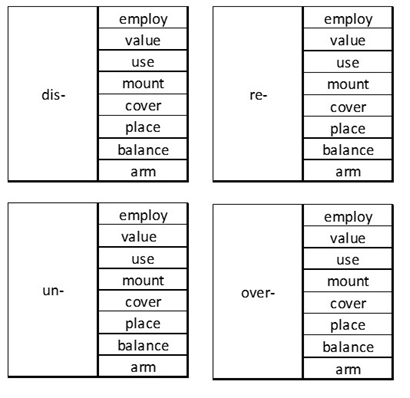


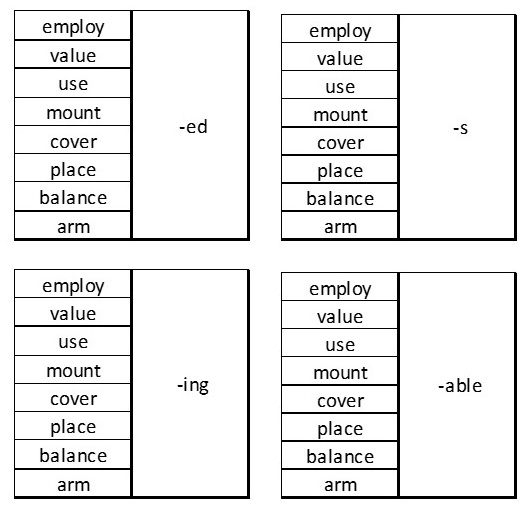


Base-centric condition


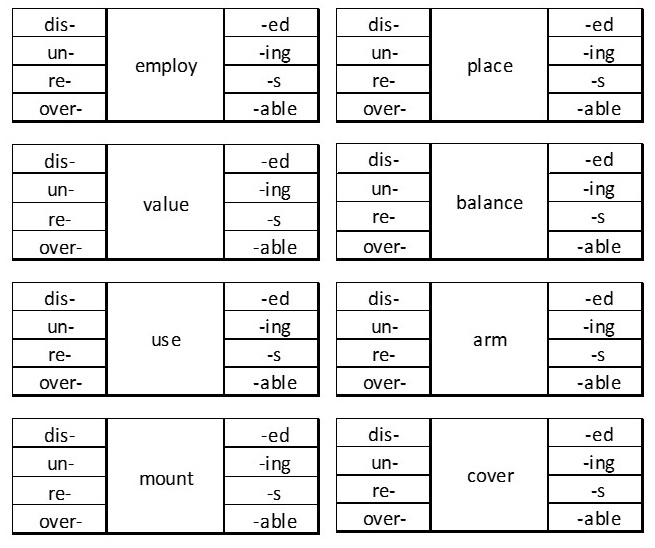


**Appendix 3 Common errors made in Experiments 1 and 2**

**Common errors in Experiment 1:**

Recombined errors

- Errors which comprise a studied base and studied affix being combined to form a non-studied word (e.g., mount + s, act + ed, re + take, dis + cover; affixes and words that were studied, but were not combined to form the target words for the recall phase).
- Examples: mounts, acted, disbalance, rewrite, charger, recount

Partial errors

- The affix “mis” was commonly used in place “dis”, and “under” instead of “un”.
- Examples: disbalance, undertake, employability

Unrelated errors

- Words like “intake”, where neither “in” nor “take” were a studied base or affix, respectively
- Examples: intake, dishonest

**Common errors in Experiment 2:**

Partial errors

- The affix “mis” was commonly used in place of “dis”, “de” instead of “dis”
- Examples: demount, misuse, misplace

Spelling errors

- “Balancable” instead of “Balanceable”
- “Placable” instead of “Placeable”
- “Valueable” instead of “Valuable”

Unrelated errors

- Examples: dishonest, honest

**Appendix 4 Word Frequencies**

| acting | 16,012 | 9.681 |
| --- | --- | --- |
| acts | 19,423 | 9.874 |
| armed | 12,786 | 9.456 |
| arming | 543 | 6.297 |
| arms | 32,708 | 10.395 |
| balanced | 10,049 | 9.215 |
| balances | 1,250 | 7.131 |
| balancing | 2,420 | 7.792 |
| chargeable | 92 | 4.522 |
| charging | 5,142 | 8.545 |
| counted | 5,341 | 8.583 |
| counter | 16,309 | 9.699 |
| covered | 25,331 | 10.14 |
| covering | 10,313 | 9.241 |
| covers | 19,777 | 9.892 |
| disarm | 991 | 6.899 |
| discharge | 2,885 | 7.967 |
| discount | 12,560 | 9.438 |
| discover | 11,099 | 9.315 |
| dismount | 371 | 5.916 |
| displace | 369 | 5.911 |
| disuse | 127 | 4.844 |
| employable | 99 | 4.595 |
| employed | 6,857 | 8.833 |
| employer | 27,757 | 10.231 |
| employing | 1,501 | 7.314 |
| employs | 1,691 | 7.433 |
| miscount | 10 | 2.303 |
| mistake | 25,420 | 10.143 |
| mounted | 8,934 | 9.098 |
| mounting | 4,678 | 8.451 |
| mounts | 2,063 | 7.632 |
| overact | 20 | 2.996 |
| overarm | 13 | 2.565 |
| overbalance | 11 | 2.398 |
| overtake | 403 | 5.999 |
| placed | 27,615 | 10.226 |
| places | 47,580 | 10.77 |
| placing | 9,529 | 9.162 |
| react | 5,926 | 8.687 |
| rearm | 66 | 4.19 |
| recharge | 835 | 6.727 |
| recover | 6,651 | 8.803 |
| remount | 239 | 5.476 |
| replace | 27,530 | 10.223 |
| reuse | 2,509 | 7.828 |
| revalue | 13 | 2.565 |
| taker | 659 | 6.491 |
| unarm | 6 | 1.792 |
| unbalance | 163 | 5.094 |
| uncover | 941 | 6.847 |
| useable | 1,308 | 7.176 |
| used | 365,662 | 12.809 |
| uses | 65,199 | 11.085 |
| using | 339,069 | 12.734 |
| valuable | 15,458 | 9.646 |
| valued | 2,741 | 7.916 |
| values | 34,017 | 10.435 |
| valuing | 268 | 5.591 |
| writer | 22,933 | 10.04 |

Words without frequency information on the ELP

| acting |
| --- |
| armable |
| balanceable |
| coverable |
| disbalance |
| disemploy |
| disvalue |
| misbalance |
| miswrite |
| mountable |
| overcover |
| overemploy |
| overplace |
| overuse |
| overvalue |
| placeable |
| rebalance |
| reemploy |
| takeable |
| unemploy |
| unmount |
| unplace |
| unuse |
| unvalue |
| unwrite |
| writable |
